# Supplementary material for: Experimentally guided models reveal replication principles that shape the mutation distribution of RNA viruses
Source: eLife. 2015 Jan 30;4:e03753. doi: 10.7554/eLife.03753 (PMC4311501; doi:10.7554/eLife.03753)
Supplement: Figure 3—source data 1. — Representative parameter sets (sets 1–5) used in Figure 3. Note that natural log values are provided for all parameters except cstay. DOI: http://dx.doi.org/10.7554/eLife.03753.006 [file elife03753s001.docx]

|  | c_trans_ | c_com_ | c_circ_ | c_rep+_ | c_rep-_ | c_pack_ | com_max_ | rep_max_ | c_3A_ | c_stay_ |
| --- | --- | --- | --- | --- | --- | --- | --- | --- | --- | --- |
| Best | -2.106 | -6.676 | -6.452 | -4.164 | 0.804 | -2.193 | 4.630 | 13.205 | 2.642 | 0.073 |
| Set 1 | -1.475 | -6.304 | -4.79 | -6.397 | 1.047 | -5.207 | 6.963 | 13.296 | 3.236 | 0.693 |
| Set 2 | -2.321 | -5.111 | -5.848 | -5.603 | 1.92 | -5.742 | 7.07 | 13.912 | 1.922 | 0.082 |
| Set 3 | -2.765 | -6.157 | -5.3 | -3.6 | 1.112 | -2.109 | 6.254 | 15.203 | 3.247 | 0.021 |
| Set 4 | -1.503 | -5.716 | -7.349 | -5.369 | 1.91 | -5.213 | 7.604 | 14.002 | 2.554 | 0.082 |
| Set 5 | -1.958 | -5.857 | -4.178 | -6.062 | 0.79 | -9.195 | 4.997 | 12.707 | 2.688 | 0.499 |
| Low | -2.027 | -5.510 | -6.636 | -5.241 | 2.191 | -5.71 | 7.233 | 14.963 | 2.222 | 0.033 |
| High | -2.163 | -5.826 | -4.639 | -5.203 | 0.934 | -9.257 | 7.042 | 14.864 | 3.765 | 0.188 |
